# Pedicled frontal periosteal rescue flap via eyebrow incision for skull base reconstruction (SevEN-002)

**DOI:** 10.1186/s12893-022-01590-3

**Published:** 2022-04-29

**Authors:** Chang Ki Jang, Soo Jeong Park, Eui Hyun Kim, Jin Mo Cho, Ju Hyung Moon, Kyoung Su Sung, Je Beom Hong, Jaejoon Joon Lim, Minkyun Na, Chang-Ki Hong, Tae Hoon Roh, Jiwoong Oh

**Affiliations:** 1 Department of Neurosurgery, Yongin Severance Hospital, Yonsei University College of Medicine, Yongin, Gyeonggi-do South Korea; 2 Department of Neurosurgery, Yonsei University College of Medicine, 50 Yonsei-ro, 120-752, Seodaemun-gu, Seoul, South Korea; 3 Department of Neurosurgery, Ewha Womans University Medical Center, Seoul Hospital, Seoul, South Korea; 4 Brain Tumor Center, Yonsei University College of Medicine, Seoul, South Korea; 5 Pituitary Tumor Center, Yonsei University College of Medicine, Seoul, South Korea; 6 Department of Neurosurgery, Catholic Kwandong University College of Medicine, Incheon, South Korea; 7 Department of Neurosurgery, Dong-A University College of Medicine, Busan, Korea; 8 Department of Neurosurgery, Sungkyunkwan University School of Medicine, Kangbuk Samsung Hospital, Seoul, South Korea; 9 Department of Neurosurgery, College of Medicine, Bundang CHA Medical Center, CHA University, Seongnam, South Korea; 10 Department of Neurosurgery, Asan Medical Center, University of Ulsan College of Medicine, 88, Olympic-ro 43-gil, Songpa-gu, Seoul, 05505 South Korea; 11 Department of Neurosurgery, Ajou University College of Medicine, Suwon, South Korea; 12 College of Medicine, Kangwon National University, Chuncheon, South Korea

**Keywords:** CSF leak, Endonasal approach, Endoscopic surgery, Pericranial flap, Skull base

## Abstract

**Purpose:**

Cerebrospinal fluid (CSF) leakage is one of the major complications after endoscopic endonasal surgery. The reconstructive nasoseptal flap is widely used to repair CSF leakage. However, it could not be utilized in all cases; thus, there was a need for an alternative. We developed a pericranial rescue flap that could cover both sellar and anterior skull base defects via the endonasal approach. A modified surgical technique that did not violate the frontal sinus and cause cosmetic problems was designed using the pericranial rescue flap.

**Methods:**

We performed 12 cadaveric dissections to investigate the applicability of the lateral pericranial rescue flap. An incision was made, extending from the middle to the lateral part of the eyebrow. The pericranium layer was dissected away from the galea layer, from the supraorbital region towards the frontoparietal region. With endoscopic assistance, the periosteal flap was raised, the flap base was the pericranium layer at the eyebrow incision. After a burr-hole was made in the supraorbital bone, the pericranial flap was inserted via the intradural or extradural pathway.

**Results:**

The mean size of the pericranial flap was 11.5 cm × 3.2 cm. It was large enough to cross the midline and cover the dural defects of the anterior skull base, including the sellar region.

**Conclusion:**

We demonstrated a modified endoscopic technique to repair the anterior skull base defects. This minimally invasive pericranial flap may resolve neurosurgical complications, such as CSF leakage.

## Background

Recently, there has been a rapid advancement in the surgical approaches exploring the anterior skull base. Akin to this advancement, many minimally invasive techniques and endoscopic endonasal approaches have been developed [3, 7, 17, 18, 26]. Particularly, the endoscopic endonasal approach is widely used to remove tumours of the sellar region, such as pituitary adenoma or craniopharyngioma. In addition, this approach is utilized to remove tumours of the anterior skull base [8, 19]. Although this technique is less invasive, it has limited application due to complications, such as cerebrospinal fluid (CSF) leakage. Thus, there have been many attempts to overcome this limitation [5, 6, 15]. In 2006, Hadad reported a nasoseptal flap technique to reduce the chances of CSF leakage [15]. Although with the use of this flap method, the problems associated with CSF leakage were resolved, the technique could not be utilized with all types of endoscopic endonasal approaches. For example, it was impossible to use the nasoseptal flap in cases with CSF leakages at the anterior skull base, an injury to the flap itself, or in those with repositionable difficulties due to poor flap patency complicated by re-operation or radiotherapy. To overcome this complication of CSF leakage during the endonasal approach, we devised a new modified pericranial rescue flap technique through cadaveric simulations.

## Methods and materials

In this study, 12 cadaveric dissections were performed to investigate the feasibility of the lateral pericranial flap technique via left eyebrow incision. The study protocol was approved by the ethics committee of our institution, and informed consent was obtained from the donors and their families. All the required consent was signed at the surgical simulation center of our institution. By abiding by the domestic law related to the cadaveric donation, the consents were directly received from the patients who volunteered for the donation before their death.

## Cadaveric procedures

### Skin incision and periosteal layer dissection

**Fig. 1:**
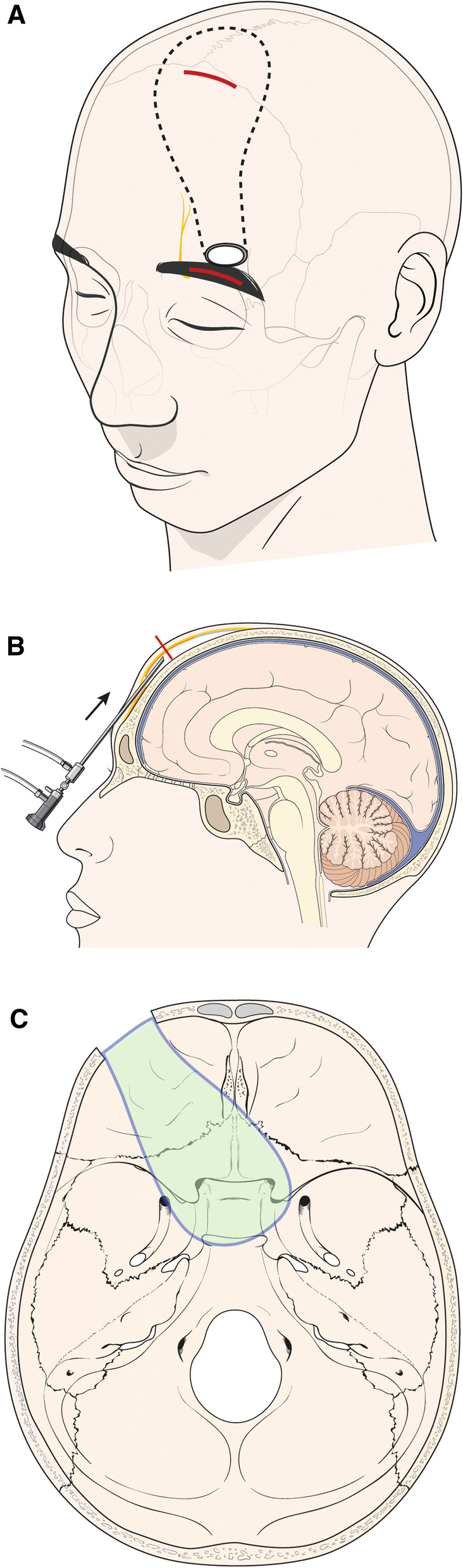
Schematic diagram of the surgical procedure. **a**, **b** The medial margin of the skin incision was at the supraorbital notch. From the supraorbital notch, about 3 cm incision was considered sufficient for the pericranial flap. After skin retraction, the periosteal layer was dissected superiorly under endoscopic view. An additional incision was made at the point where dissection was not possible due to the natural skull curvature (red line). **c**, **d** Through the burr-hole on the supraorbital area, the pericranial flap was inserted in the medial direction via the extradural or intradural pathway. The pericranial flap could cover the sellar and anterior skull base defects

**Fig. 2:**
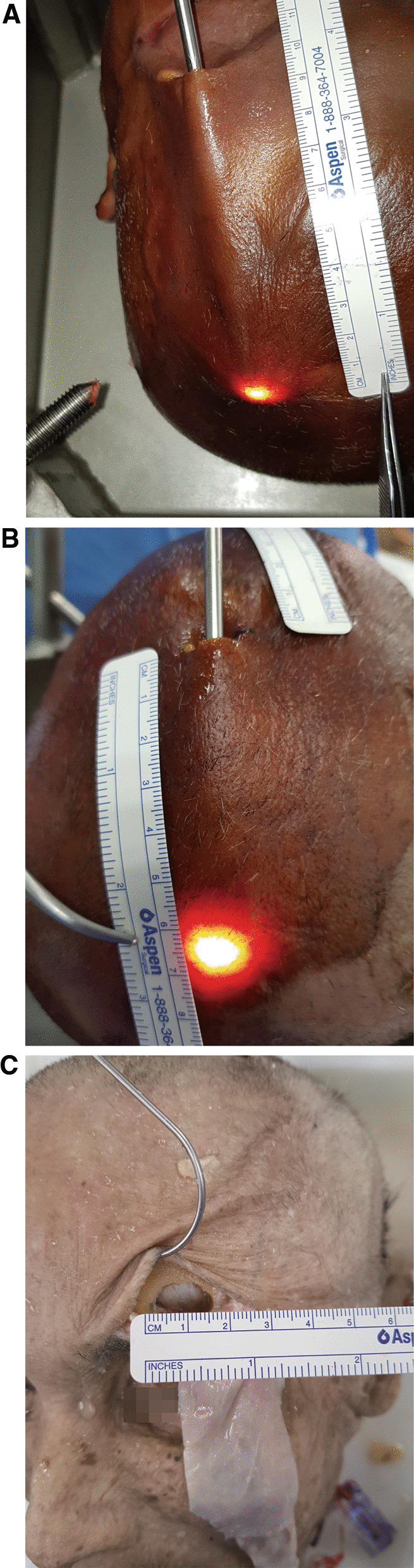
Schematic procedure for additional incision and burr-hole. **a** Approximately 10 cm superiorly from the eyebrow incision, further periosteal dissection was impossible due to the natural shape of the skull. **b** At this point, we made an additional incision. Through this additional incision, we could harvest a longer periosteal flap. **c** After a periosteal incision with a No. 12 blade, we pulled out the periosteal flap via the eyebrow incision. **d** We made a single burr-hole on the supraorbital area. Then, we inserted the pericranial flap via the intradural or extradural pathway

The head of the cadaver was positioned with a slight extension. The skin incision was placed within the eyebrow range with the medial margin of the incision at the supraorbital notch. A 3 cm incision was made in the lateral direction, beginning at the supraorbital notch. A 2.5–3 cm incision was considered sufficient to provide access to the surgical field. After the skin incision, the skin was retracted superiorly, and the galea aponeurotica was dissected from the pericranium using a Metzenbaum scissor. The integrity of the layer was checked by an endoscope from time to time (Fig. 1). While dissecting the scalp with Metzenbaum scissor via the eyebrow incision, when further dissection was no longer possible due to the natural curvature of the skull, an additional incision of 1–2 cm was made on the scalp behind the hairline. Through the additional incision, the operator verified that the pericranial layer was dissected from the eyebrow region and, subsequently, moved towards the occipital side to further dissect the pericranium layer (Fig. 2). Next, an incision was made on the pericranium layer using the No. 12 blade. The pericranium layer was detached from the skull using a dura elevator, and the flap was pulled out through the eyebrow incision.

### *Making a burr-hole and inserting a flap *via* extradural or intradural pathways*

**Fig. 3:**
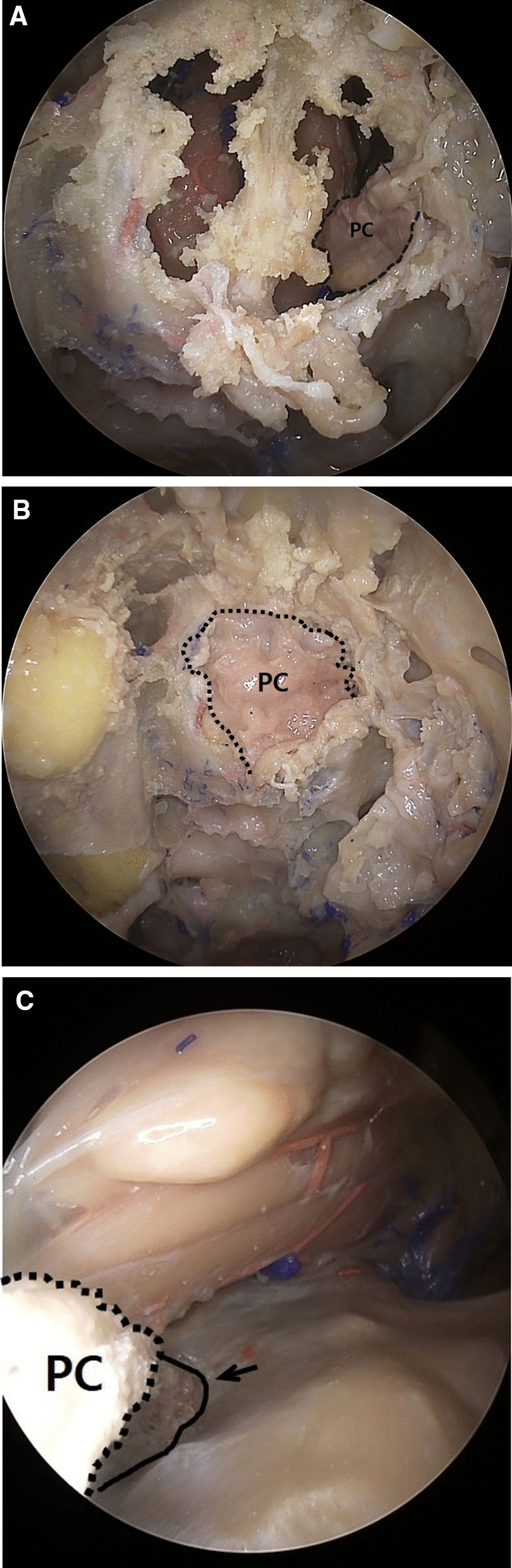
Repositioning of the pericranial flap (PC) via the endonasal endoscopic approach. **a** A flap inserted through the supraorbital burr hole is partially visible on the margin of a pre-made cribriform defect (endonasal view). **b** Flap repositioning was done via endonasal endoscopic approach. As a result, the cribriform plate defect was fully covered (endonasal view). **c** Endoscope view via a supraorbital burr hole. Pedicled pericranial flap inserted to cover the cribriform plate defect (arrow)

**Fig. 4:**
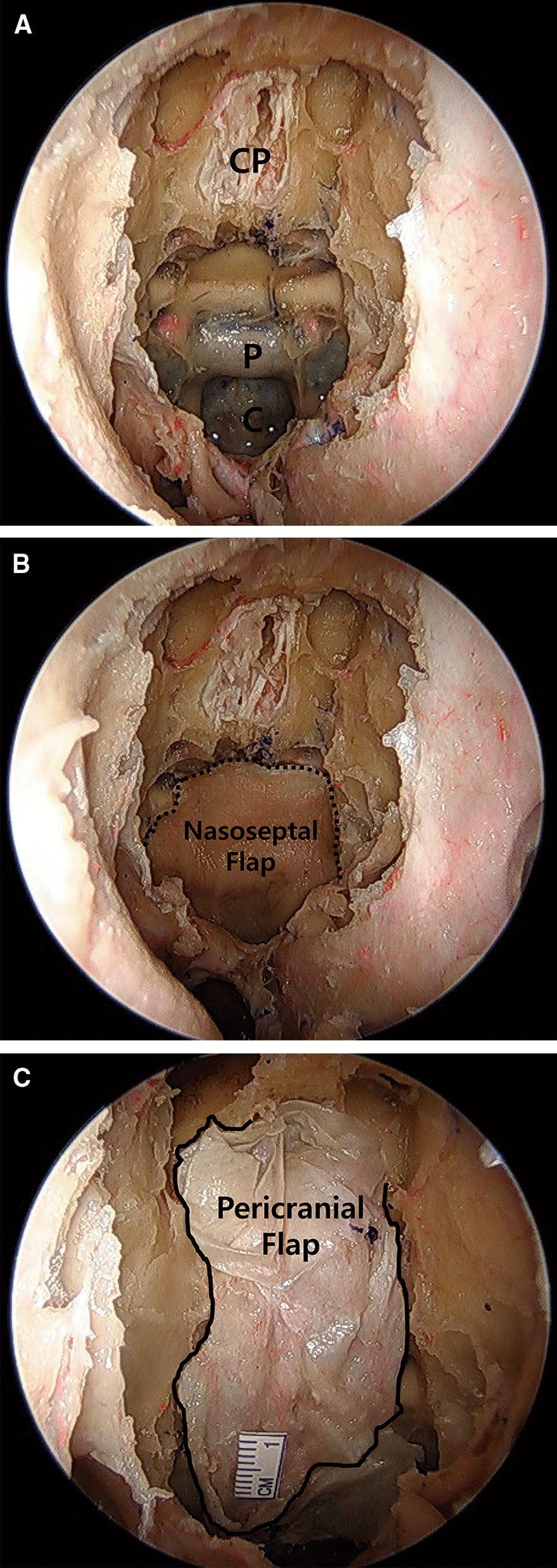
Endoscopic endonasal view of the anterior skull base. **a** Dural defect area before pericranial flap placement. The dura defect on the cribriform plate area was pre-made prior to the periosteal flap harvest. CP, cribriform plate; P, pituitary region; C, clivus. **b** Using this nasoseptal flap, an attempt was made to cover the dural defect in the cribriform plate. The end of the nasoseptal flap could not reach the cribriform plate area. **c** Using our lateral pericranial flap method, the flap could fully cover the anterior frontal base, sellar, and clivus regions

While maintaining the superior retraction at the initial incision site, a burr-hole with a diameter of 2 cm was made at the base of the frontal bone above the orbit (i.e., at an area superolateral to the supraorbital notch), avoiding the supraorbital nerve branching out from the medial side of the supraorbital notch. Care should be taken to avoid damage to the pericranial flap while making a burr-hole. After the dura exposure, the dura incision was either made through the extradural or intradural pathway, based on the surgical plan. When the flap was inserted via the extradural pathway, the dural flap was dissected using a dura elevator from the burr-hole site towards the sellar and cribriform plate, retracting the dural layer in the superior direction. Then, the flap was held by cup forceps and inserted in the direction of the sella turcica using an endoscope. Whereas, when the flap was inserted via the intradural pathway, the brain was retracted in the superior direction, and the flap was inserted in the direction of the sella turcica. Final repositioning of the flap was done while observing through an endoscopic endonasal view; the aim at this stage of the operation was to insert the flap until it partially covered the bony defect area at the sellar region and the anterior skull base (Fig. 3, 4).

### *Confirmation of pericranial flap position at the dural defect site *via* endonasal endoscopic view*

In some cadavers, we deliberately made bony and dural defects at the sellar and cribriform plate regions prior to cadaveric surgical simulations via the endoscopic-endonasal approach. The pericranial flap was partially inserted at the burr hole site through the eyebrow incision, and the final repositioning was done using cup forceps under the endonasal endoscopic view. Then, we verified whether using a pericranial flap, it was possible (1) to cover the sellar area and frontal defect; (2) to approach via the extradural and intradural pathways; and (3) to cross the midline. A pericranial flap was considered successful if it could fully cover the dural defect and was considered reachable if it covered the clivus (Fig. 4)**.** Technical failure was defined as follows: (1) the pericranial flap derived from the eyebrow incision could not reach the bony defect at the sellar and frontal base regions; or (2) the flap could not fully cover the dural defect.

## Results

We simulated this new technique through 12 cadaveric dissections. Of 12 trials, we failed in the initial four surgical trials. However, we managed to re-evaluate and resolve troubleshooting problems to succeed in the rest of the eight trials. Except for one case, in which the pericranial flap was too thin, we skillfully performed the procedure in the rest of the seven trials. The complications, such as frontal sinus opening, were not observed as the flap was medially and diagonally directed from the burr-hole, which was located lateral to the supraorbital notch.

### Analysis of failed cadaveric cases

Initially, we utilized the maximal pericranial flap, acquired from the eyebrow incision site, without an additional incision at another anatomical site. However, the flap with an average length of 5.83 cm, harvested by the initial method, was too short to cover the sellar region and the frontal base. Thus, as mentioned earlier, the initial four cadaveric trials failed due to the generation of short flaps via only one surgical incision at the eyebrow (Table 1). Of note, flap length was even shorter when the supraorbital notch was positioned too lateral in cadavers. We confidently succeeded to perform seven cadaveric simulations with an additional incision behind the hairline to acquire an extra flap length to cover the defect. Meanwhile, one case failed even with the same trial because the cadaveric skin, as well as the flap, was too thin.

**Table 1:** Failure cases to obtain proper pericranial flap

Case number	Length of flap (cm)	Additional incision	Reason of failure
1	5.5	No	Short flap (Technical limitation arisen from no additional incision on the scalp)
2	5.9	No	Short flap (Technical limitation arisen from no additional incision on the scalp)
3	6.1	No	Short flap (Technical limitation arisen from no additional incision on the scalp) The location of supraorbital notch was too lateral
4	5.8	No	Short flap (Technical limitation arisen from no additional incision on the scalp) Supraorbital nerve injury (Retraction injury)
5	11.1	Yes	Skin was too thin (In the process of dissection, the skin penetration and flap disconnection was happened. Therefore, we could not attain the flap through periosteal dissection)

### Analysis of successful cadaveric cases

Using the modified technique with an additional incision, we successfully acquired a sufficient pericranial flap with an average length of 11.53 cm in seven of the eight cadaveric trials. After the acquisition of the flap, it was possible to insert it via both extradural and intradural pathways. As a result, the flap could sufficiently cover the defects, including the frontal base, cribriform plate, and sellar regions (Table 2). Moreover, we could cross the midline with the flap in the cadavers with deliberate defects at the frontal base and cribriform plate, which were made before the cadaveric surgical simulations. On the contrary, we could not cross the midline while inserting the flap in the cases without deliberate bony defects because of the anatomical obstruction at the falx cerebri and crista galli.

**Table 2:** Successful cases to obtain proper pericranial flap with additional skin incision

Case number	Length of flap (cm)	Additional incision	Intradural insertion	Extradural insertion	Coverage of sellar region	Coverage of frontal base
1	11.3	Yes	Possible	Possible	Fully covered	Fully covered
2	11.8	Yes	Possible	Possible	Fully covered	Fully covered
3	12.1	Yes	Possible	Possible	Fully covered	Fully covered
4	11.2	Yes	Possible	Possible	Fully covered	Fully covered
5	11.4	Yes	Possible	Possible	Fully covered	Fully covered
6	11.3	Yes	Possible	Possible	Fully covered	Fully covered
7	11.6	Yes	Possible	Possible	Fully covered	Fully covered

## Discussion

In general, the dural defect resulted during the endoscopic endonasal approach can be overcome with nasoseptal flaps. However, it can be difficult to apply nasoseptal flaps in the cases with the tumor invasion in the nasospetal region or in the special surgical situations with the septal flaps which are too short to cover the dura defect of anterior cranial fossa.

In this study, we attempted to acquire a rescue flap targeting this patient group [24, 27]. Therefore, the aim of the study was to design a surgical method to obtain a pericranial flap that does not result in CSF leakage, which is one of the major complications during endoscopic endonasal surgery. Furthermore, we could demonstrate that our novel surgical technique has three advantages: (1) unlike the method by G. Hadad, the pericranial flap acquired by our method can cover the defects in the frontal base and the cribriform plate region; (2) no anatomical structures in the frontal sinus are violated except in the case of a large frontal sinus; and (3) cosmetic aspect is taken into consideration. In brief, an intact pericranial flap was obtained to cover the sellar region and the frontal base by making a burr-hole lateral to the supraorbital notch without damaging the frontal sinus region. In addition, we minimized cosmetic problems by using an eyebrow incision.

Recently, the endoscopic endonasal approach for removing the skull base tumours is rapidly gaining popularity because of its advantages in providing a wide surgical view with minimal invasiveness [11, 23, 25]. Even the cases with extensive involvement, including tuberculum sella, anterior cranial fossa (such as cribriform plate and olfactory groove), Meckel’s cave, and pterygoid fossa, can be approached using this technique [1, 9, 16]. However, since the surgery involves the bone and dura in the skull base, CSF leakage has been the main concern with this approach. In the trans-sphenoidal surgeries, including the endoscopic endonasal approach, the incidence rate of CSF leakage was reported to be approximately 1.3–6% [4, 14]. Forbes et al. [11] reported postoperative CSF leakage in 10% (1/10) of the cases after the endonasal endoscopic transsphenoidal resection of intrinsic third ventricular craniopharyngioma. Several surgical techniques and materials have been utilized to reconstruct the skull base and prevent CSF leakage in clinical settings [10, 12, 13, 20]. Among them, the most widely used material is the nasoseptal flap.

Owing to the rapid advancements in the development of nasoseptal flaps since 2006, the incidence rate of CSF leakage after the endoscopic endonasal surgery has decreased from 50 to 5% [15]. Since then, the pedicled nasoseptal flap has become mandatory for the reconstruction of skull base defects. Nasoseptal flap has several advantages. Firstly, the flap can be obtained without any external surgical incision. Secondly, the flap is viable and more stable than other non-pedicled flaps as it receives its blood supply from the sphenopalatine artery. Thirdly, the flap is flexible and readily available; it can easily be extended in width [21]. However, the nasoseptal flap has several disadvantages as well. The removal of the septal mucosa may result in complications, such as nasal septal perforation, cartilage necrosis, and nasal deformity. Furthermore, there may be short-term impairment in nasal mucociliary clearance [2]. Of note, the pedicle for the nasoseptal flap begins from the coanas and ostium; hence, it is often impossible to extend the flap until the frontal base.

The use of the pericranial flap in the skull base surgeries was first reported in 2009 by Zanation et al. [27]. They published a case report on a brain tumour involving the right ethmoidal sinus. They made a surgical incision on the glabella and inserted the pericranial flap directly through the midline. However, besides advantages, using the glabella incision, they reported a risk of opening in the frontal sinus and a cosmetic dilemma. Therefore, we developed a pericranial flap which overcame these disadvantages. Here, we restricted the first incision to the eyebrow and placed it in a lateral-to-medial direction to avoid any injury to the frontal sinus; the flap was named as the lateral pericranial rescue flap. Although there was no evidence of frontal sinus invasion in our study, it is essential to obtain a computed tomography scan or radiograph before surgery as some patients may have a laterally positioned frontal sinus.

The pedicled frontal periosteal rescue flap may be recommended when either the nasoseptal flap cannot be harvested or is short enough to cover the anterior cranial fossa (cribriform plate, planum sphenoidale).

Although in this study, there was no discontinuity in the olfactory nerve, the olfactory nerve may get injured due to frictional force during positioning of the flap in the anterior skull base close to the cribriform plate. However, with the advancement and positioning of the rescue flap via the endonasal route, the probability of direct retraction and manipulation of the brain parenchyma is less (Fig. 3).

### *Application of the pericranial flap *via* intradural or extradural pathways*

In this cadaveric study, we inserted the pericranial flap through a burr-hole in the supraorbital area. The degree of difficulties encountered while inserting the flap through intradural and extradural pathways were different in cadavers without prior bony and dural defects at the sellar region and frontal base. There was a higher risk of dural tear while detaching the dura from the frontal base for flap insertion via the extradural pathway; particularly, it was difficult to detach the dura from the cribriform plate. In contrast, because of the falx cerebri, it was impossible to insert the flap in the anterior fossa by crossing the midline via the intradural pathway; besides, there was limited surgical space required to cut the falx cerebri and retraction of brain parenchyma with simultaneous insertion of the flap was not easy.

However, when we simulated clinical conditions (such as osteolysis of the frontal base due to tumour mass or dura detachment/elevation at the cribriform plate due to surgical damage) by deliberately making bony and dural defects in the sellar region and the frontal base prior to the actual surgical repair using the pericranial flap, no surgical difficulties were encountered with either intradural or extradural approach. Except, there was a risk of mechanical damage to the brain cortical surface while advancing the flap through the burr hole via the intradural approach. Besides, there could be CSF leakage because of an additional dura incision. Therefore, a rescue flap and artificial dural reinforcement may be needed for the prevention of CSF leakage.

### Limitations

This study has certain limitations. The size of the sample, comprising cadaveric cases, was small. Moreover, we could not replicate the actual clinical conditions due to the non-viability of the tissues in cadaveric specimens. Therefore, a detailed examination of the lateral pericranial rescue flap obtained using our surgical technique in clinical settings is imperative.

In addition, some complications may arise with this surgical flap method. Firstly, the facial nerve branches may injure during the eyebrow incision. Reisch and Perneczky [22] reported a 5.5% incidence of permanent palsy of frontalis muscle during the eyebrow incision. Secondly, the eyebrow incision itself can violate the supraorbital notch causing an injury to the supraorbital nerve injury; the patients may complain about eye pain and forehead numbness. Thirdly, however less, there are chances of damage to the olfactory nerve; the olfactory nerve may injure while inserting the pericranial flap during the intradural surgical pathway. For the prevention of olfactory nerve injury and frontal sinus violation, a more lateral approach would be suitable. For a lateral supraorbital approach, a pedicled temporoparietal fascia flap may be one of the possible alternatives.

## Conclusion

This study demonstrated a novel surgical method in acquiring an intact pericranial rescue flap without violating the frontal sinus or creating cosmetic problems. This technique can be an alternative option for the surgical cases in which the nasoseptal flap by Hadad et al. [15] is not applicable during the primary repair of CSF leakage. We have not yet implemented this method in clinical settings. However, it is theoretically feasible, as shown by cadaveric simulations; thus, we believe it can be utilized in some neurosurgical cases with CSF leakage.

## Data Availability

All data generated or analysed during this study are included in this published article.

## Abbreviations

PC: Pericranial flap
CSF: Cerebrospinal fluid
